# Effect of topical fluoride on microshear bond strength of primary enamel to composite, microhardness of enamel and its surface morphology: An in vitro study

**DOI:** 10.15171/joddd.2019.046

**Published:** 2019

**Authors:** Mina Biria, Sajedeh Namaei Ghasemi, Seyedeh Mahsa Sheikh-Al-Eslamian, Narges Panahandeh

**Affiliations:** ^1^Department of Pediatric Dentistry, Dental School, Shahid Beheshti University of Medical Sciences, Tehran, Iran; ^2^Private Practice, Tehran, Iran; ^3^Dental Research Center, Research Institute of Dental Sciences, Dental School, Shahid Beheshti University of Medical Sciences, Tehran, Iran

**Keywords:** Dental enamel, fluorides, hardness

## Abstract

***Background.*** This in vitro study aimed to evaluate the microshear bond strength (μSBS), microhardness and morphological characteristics of primary enamel after treating with sodium fluoride (NaF) and acidulated phosphate fluoride (APF).

***Methods.*** Forty-eight primary canines were cut into mesial and distal sections and assigned to five groups randomly: group 1 (immersed in saliva as a control), group 2 (treated with NAF and immersed in saliva for 30 minutes), group 3 (treated with APF and immersed in saliva for 30 minutes), group 4 (treated with NAF and immersed in saliva for 10 days), and group 5 (treated with APF and immersed in saliva for 10 days). Composite resin (Filtek Z250) was bonded on the specimens (n=15) for measuring the μSBS. After storage in 37°C artificial saliva for 24 hours, µSBS and Vickers hardness tests (10 readings) were performed. The data were analyzed using one-way ANOVA and Kolmogorov-Smirnov, Levene’s and Tukey HSD tests (P<0.05). Morphological analysis of enamel and modes of failure were carried out under a scanning electron microscope (SEM) on two remaining specimens.

***Results.*** Significant differences in μSBS were only noted between groups 2 and 4 (P=0.024). Group 3 showed a significant decrease in hardness after storage in artificial saliva (P<0.001), with a significantly lower hardness than the other groups (P<0.001). The SEM observations showed irregular particles in groups 3 and 5; uniform, smooth and thin coats were seen in groups 2 and 4.

***Conclusion.*** Fluoride therapy with NaF and APF gels prior to restorative treatments had no adverse effects on the microshear bond strength.

## Introduction

Fluoride therapy is a commonly used method for caries prevention in children and adults, which also enhances remineralization.^1,2^ Different methods of fluoride therapy include daily use of fluoride mouthwashes, application of fluoride gels and use of fluoride varnishes in the dental office.^3,4^

In pediatric dentistry, fluoride therapy is often performed as the first treatment procedure in the first dental visit of patients and prior to other procedures. In such cases, the possible adverse effects of fluoride therapy on the properties of restorative materials are often a concern for many clinicians. It has been proposed that topical application of fluoride solutions may decrease the surface energy and reduce the wettability and flowability of adhesive materials.^5^

It was reported that the topical use of fluoride on the enamel surface prior to acid etching decreases the bond strength of fissure sealants. Thus, it is suggested that sealants not be applied after the use of topical fluoride, and sealant therapy should be carried out prior to fluoride therapy or postponed to another session.^6^ Exposure of enamel to fluoride leads to the formation of fluorapatite, which is less soluble than hydroxyapatite. Teeth with a high concentration of fluoride are often more resistant to etching and require longer conditioning time. It was reported that teeth with fluorosis have lower bond strength to orthodontic brackets compared to normal enamel.^7^ In contrast, some studies have shown that fluoridated toothpastes have no significant effects on bond strength.^8^

Meng et al^9^ found that the application of APF to tooth structure after etching decreased the bond strength of the bracket to the enamel. On the other hand, Kimura et al^8^ evaluated the effect of fluoride varnish on the bond strength of orthodontic brackets using self-etch and etch-and-rinse adhesives and reported that NaF varnish did not have any significant effect on the bond strength of orthodontic brackets bonded 10 days later. Thus, this study aimed to assess the effect of topical fluoride therapy with NaF and APF on the µSBS of immediate and delayed composite resin restorations to primary enamel. The microhardness of enamel after fluoride therapy was also measured, and the treated enamel surfaces were inspected under a scanning electron microscope to assess the effect of the two fluoride therapy protocols on enamel morphology.

## Methods

Table 1 presents the characteristics of the materials used in this study. Forty-eight extracted sound human primary canines with a minimum of two-thirds of the root length remaining were used. The teeth were cleaned with pumice to remove any surface debris and stored in 0.5% chloramines T solution (4°C, 24 hours) before the experiment. The teeth were cut into mesial and distal pieces, and the roots were cut 2 mm under CEJ, using a diamond disc (D & Z, Berlin, Germany). Each specimen was treated with 600-grit silicone paper in order to obtain a flat enamel surface. The specimens were randomly divided into five groups, as follows (n=19):

**Table 1:** The characteristics of the materials used

**Material**	**Characteristic**	**Compositions**	**company**
Alpha Etch GEL	Etching gel	37% phosphoric acid	NOVA DFL Brazil
Adper^TM^Single Bond	One-bottle total-etch adhesive	BisGMA, HEMA, dimethacrylates, ethanol, water, a novel photoinitiator system and a methacrylate functional copolymer of polyacrylic and polyitaconic acids	3M ESPE Dental products St. Paul, MN, USA
Filtek^TM^Z250	Universal Restorative A_1_ Shade	Zirconia/silica Inorganic filler loading is 60% by volume (without silane treatment) with a particle size range of 0.01 to 3.5 µm. BIS–GMA, UDMA, BIS-EMA	3M ESPE Dental Products, St. Paul, MN, USA
KIN hydrate	Artificial saliva Spray for dry mouth	Xylitol, potassium chloride, sodium chloride, calcium chloride, Magnesium chloride, potassium Dihydrogen phosphate, potassium thiocyanate, sodium saccharin, other excipients.	LABORATORIOS KIN S.A. E-08018 Barcelona-Spain
Topex	Topical APF gel Thixotropic (PH 3.5)	Acidulated phosphate Fluoride containing 1.23% Fluoride ion	Sultan USA
Neutral Fluoride Preventive Treatment Gel	NaF (pH=7)	2% sodium fluoride (0.9% fluoride ion)	Pascal USA

**Group 1:** The specimens were immersed in artificial saliva at 37°C for 30 minutes (Kin Hidrate, Kin, Spain) as controls.

**Group 2:** The specimens were treated with 2% NaF gel according to the manufacturer’s instructions and stored similar to that in group 1.

**Group 3:** The specimens were treated with 1.23% APF gel according to the manufacturer’s instructions and stored similar to that in group 1.

**Group 4:** The specimens were treated similar to that in group 2 and immersed in artificial saliva for 10 days.

**Group 5:** The specimens were treated similar to that in group 3 and immersed in artificial saliva for 10 days.

After removal from the artificial saliva, the samples were rinsed under running water for 15 seconds and dried. Acid (Alpha Etch GEL NOVA DFL, Brazil) and Single Bond adhesive (3M/ESP, USA) were applied on the specimens (n=15 in each group) according to the manufacturer’s instructions. A micro Tygon tube with an internal diameter of 0.7 mm and a height of 1 mm was placed on the enamel surface and filled with composite resin. Light-curing was performed for 40 seconds (Demetron LC, Kerr, Orange, CA, USA). The specimens were immersed in 37°C artificial saliva for 24 hours, and the tube was removed before the μSBS test.

### Microshear bond strength (μSBS) measurements

For μSBS measurement, the specimens were fixed on a microtensile testing machine (Bisco, NJ, USA) using a special apparatus. A shear force was applied to the composite resin‒enamel interface at a crosshead speed of 1 mm/min until fracture. The μSBS values were calculated in MPa using the following formula: F/лr^2^, where F is the load at fracture in N, and r is the radius of the cross-section.

### Failure modes 

After the μSBS test, the fracture surface of each specimen was observed under a stereomicroscope (SEM) (Topcon ABT 150S, Topcon Co., Tokyo, Japan) to record the failure mode. Failure modes were classified as adhesive failure between the enamel and resin, cohesive failure within the composite resin or mixed failure.

### Microhardness measurements

Two specimens in each group were used for Vickers microhardness measurement. After treatment, the specimens were mounted in cylindrical molds containing autopolymerizing acrylic resin. A Vickers microhardness tester (HVS-100 Digital Display Hardness tester, Laizhou, Shandong, China) was used for microhardness measurement, and the indenter applied a 100-g load to five different points in each sample for 10 seconds (10 points in each group). The mean microhardness number was calculated for each group.

### Morphological surface analysis

Two specimens were used in each group for SEM assessments. After fluoride therapy, the specimens were placed in an oven to dry. The specimens were then gold sputter-coated and evaluated under an SEM (Topcon ABT 150S, Topcon Co., Tokyo, Japan) at ×15000 magnification.

### Statistical analysis

The Kolmogorov-Smirnov test was used to assess the normal distribution of data. Equality of variances in the groups was tested with Levene’s test. One-way ANOVA was used to detect any significant differences in μSBS and microhardness between the groups. Pair-wise comparisons were carried out with the Tukey HSD test (P<0.05).

## Results

Normal distribution of the data was ensured in all the five groups by one-sample Kolmogorov-Smirnov test (P=0.563). Equality of variances was also confirmed using Levene’s test (P=0.37). Thus, one-way ANOVA was used to compare the groups.

The mean μSBS values of all the groups are presented in Table 2. The results showed significant differences in μSBS values between the groups (P<0.05). Pair-wise comparisons revealed a significant difference in μSBS between groups 2 and 4 (21.52±2.04 MPa vs. 29.93±1.57 MPa; P=0.024). Although the mean µSBS values of groups 2 and 3 were lower than that of the controls, this difference did not reach statistical significance. In addition, groups 4 and 5 showed a bond strength value even higher than that of the control; but this difference was not statistically significant either. It was found that the majority of fractures were mixed (adhesive‒cohesive). Adhesive failure had a higher frequency than cohesive failure (Table 2).

**Table 2:** The mean μSBS values, modes of failure and microhardness values in all the groups

**Group**	**µSBS (MPa)**†**M ean ± SE**	**Mode of failure**			Hardness (VHN)*Mean ± SD
		**Adhesive (%)**	**Cohesive (%)**	**Mixed (%)**	
**1**	25.01±1.87	5 (33.3)	0 (0)	10 (66.7)	^A^314.50±24.95
**2**	^a^21.52±2.04	4 (25.0)	2 (12.5)	9 (60.0)	^A^290.94±38.61
**3**	23.93±1.91	3 (20.0)	2 (13.3)	10 (66.7)	242.50±28.87
**4**	^a^29.93±1.57	2 (13.3)	1 (6.7)	12 (80.0)	^A^320.98±30.97
**5**	26.93±2.18	3 (20.0)	2 (13.3)	10 (66.7)	^A^284.90±38.53

†Values with the same letter are significantly different. *Values with the same letter are not significantly different.

Normality of the microhardness data (Table 2) was ensured using Kolmogorov-Smirnov test (P=0.230). Equality of variances was also confirmed by Levene’s test (P=0.479). Thus, one-way ANOVA was applied for multiple comparisons of the mean microhardness values, which showed a significant difference between the five groups (P<0.001). Pair-wise comparisons by Tukey HSD test revealed that groups 1, 2, 4 and 5 were not significantly different but group 3 had a significantly lower hardness number than the other four groups. Although the remaining four groups were not significantly different in terms of hardness number, group 5 had the lowest (284.9±38.53) and group 4 had the highest (320.9±30.97) VHN.

### Morphological surface analysis 

**Figure 1:**
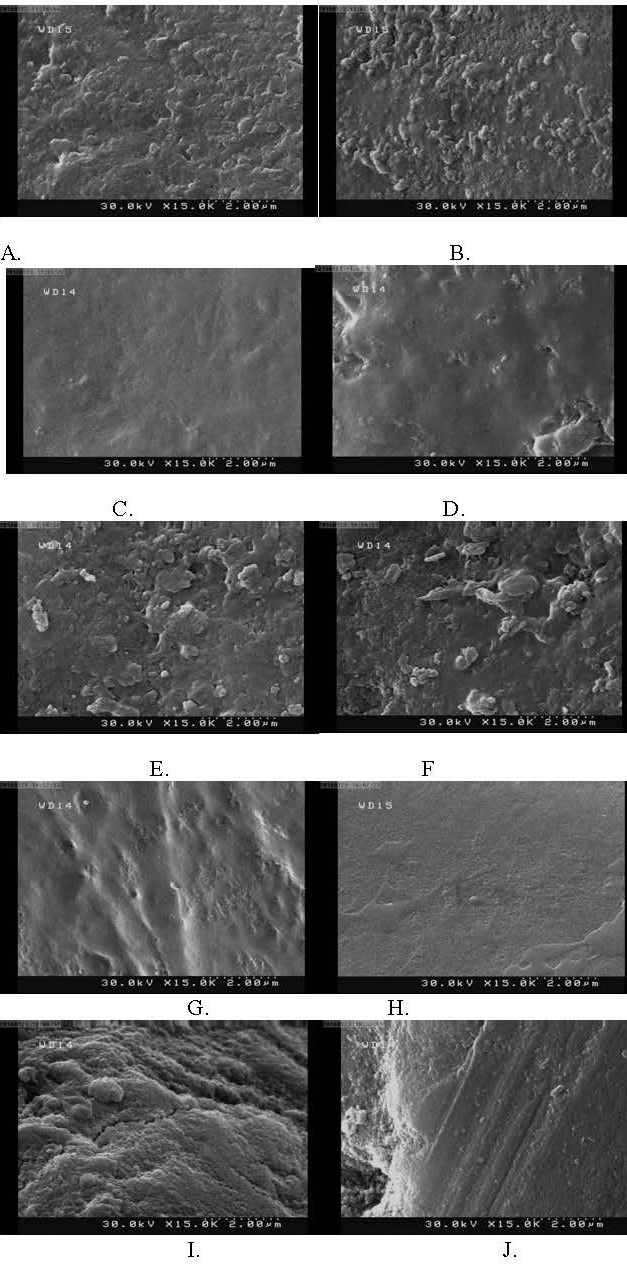
The scanning electron microscopic micrographs; (A, B) immediate APF; (C, D) immediate NaF; (E, F) delayed APF; (G, H): delayed NaF and (I, J) control group.

In group 1, grooves were observed on the enamel and the smear layer was the only phenomenon seen on the enamel surface. In groups 3 and 5, particles were noted on the enamel surface, which could be due to the effect of APF and deposition of fluoride on the enamel surface. In other words, fluoride therapy with APF caused irregularities on the enamel surface and made the enamel surface coarser. In groups 2 and 4, deposition of particles was not seen; instead, a uniform, thin coat was observed that covered the entire enamel surface, which might have been responsible for a stronger bond and higher hardness value. The NaF groups had smoother surfaces with lower surface roughness (Figure 1).

## Discussion

Fluoride therapy is one of the most common components of caries prevention protocol in children and adults. Considering the increasing demand for tooth-colored restorations, the effect of fluoride therapy on the bond strength of restorative materials to enamel has always been a concern. No previous comprehensive study has compared the possible effects of APF and NaF fluoride gels on the bond strength of composite resin to enamel and the most suitable time for restoring primary teeth following fluoride therapy. Therefore, this study compared the effects of 1.23% APF and 2% NaF gels on the µSBS of composite resin to primary enamel when restoring the teeth immediately or 10 days after fluoride therapy and on the microhardness of treated enamel.

Our findings showed a decrease in the bond strength of composite resin to primary enamel immediately after fluoride application. However, this reduction was not significant, consistent with the findings of other investigations.^10-14^ It has also been demonstrated that the bond strength of sealants to enamel is not influenced by fluoride therapy.^15,16^ In this regard, the current study revealed that fluoride therapy had no adverse effect on the bond strength of composite resin to enamel. Thus, it was concluded that fluoride therapy can be immediately followed by fissure sealant therapy or restorative treatments in the same session. Bahrololoumi et al^11^ evaluated the effect of the application of topical fluoride (APF gel) on the µSBS of fissure sealants to enamel and reported no significant differences between the test and control groups. Based on their findings, fluoride therapy can be performed before fissure sealant therapy in one single session.

The current findings showed that postponing composite resin restorations for 10 days after fluoride therapy with 2% NaF gel increased the bond strength to a level higher than that of the controls. Similarly, Leodido et al^17^ reported that the control group had significantly higher bond strength than groups treated with APF and SFV (sodium fluoride varnish). They also showed that fluoride therapy with NF (neutral fluoride gel), immediately before bracket placement, had no adverse effects on the bond strength and resulted in no significant difference from the control group.

Choi et al^18^ inspected the samples by atomic force microscopy and SEM and found that application of APF decreased the surface roughness of enamel during etching and recommended postponing tooth-colored restorations for two weeks after fluoride therapy to obtain maximum retention and bond strength.^18^ Based on our findings, the bond strength in samples restored after 10 days following fluoride therapy was even higher than that of the control group, but this difference was only significant in the NaF group. Thus, in case of fluoride therapy with NaF, restorative treatments can be postponed for 10 days to achieve maximum bond strength.

In contrast to our findings, Low et al^19^ showed that treatment of the etched enamel with APF decreased the bond strength of five different sealants, which might be due to the formation of reactive products, which appear in the form of globules on the enamel surface under SEM. The difference between their findings and ours is probably attributed to the fact that they applied APF on etched enamel, which is different from our methodology.

Gwinnett et al^20^ and Kochavi et al^21^ used SEM to assess the effect of different topical fluoride formulations, including APF on the etched enamel and observed the formation of globular structures on the etched surface due to the reaction of CaF_2_. This product can be responsible for the reduction of bond strength. Thus, thorough rinsing after fluoride therapy was recommended by authors to remove the residual product from the surface, since it might interfere with the bonding process. Based on their findings, fluoride therapy can be performed prior to fissure sealant therapy in one single session.^11^

Evidence shows that NaF gel can react with enamel hydroxyapatite and form a thick layer of calcium fluoride.^22^ A previous study showed that groups treated with APF experienced a reduction in microhardness, which might be attributed to its low pH (3.2 to 3.5);^23^ this finding was consistent with our results, in which APF significantly lowered the hardness number compared to NaF. Moreover, in the afore-mentioned study, SEM analysis revealed enamel surface irregularities and increased porosities following the application of 1.23% APF, which can decrease microhardness; these findings were also consistent with our results.

Assessment of the samples under SEM in our study revealed particles and irregularities on the enamel surface in both immediate and delayed APF groups, which were not seen in the control group. These particles were probably formed due to the effect of APF on the enamel and deposition of fluoride on the enamel surface. In other words, APF created irregularities and yielded a coarse surface. APF made the enamel surface coarser, which could be due to the effect of APF and deposition of fluoride on the enamel surface. However, APF, due to its acidity, degrades the enamel surface and adversely affects the bond strength and microhardness.

In the NaF samples, deposition of particles was not seen; instead, a uniform, thin coat covering the entire enamel surface was noted, which might be attributed to a stronger bond and higher hardness number.

The surface of samples in NaF groups seems to be smoother, with lower roughness. In the delayed NaF group, the bond strength was significantly higher than that in the other groups, which might be attributed to the presence of the afore-mentioned thin coat covering the entire enamel surface and its positive effect on bond strength and hardness. In other words, NaF probably modified the enamel surface as supported by the SEM findings.

## Conclusion

Fluoride therapy with NaF and APF gels prior to tooth-colored restorative treatments has no adverse effect on the bond strength; thus, fluoride therapy and restorative treatments can all be performed in a single session. In case of fluoride therapy with NaF, restorative treatments can be postponed for 10 days if a higher bond is desirable.

## Authors’ contributions

MB conceived the idea and designed the study. SNGh carried out the experiments. SMSAE performed the analytic calculations. NP contributed to sample preparation and wrote the manuscript. The manuscript has been read and approved by all the authors.

## Acknowledgments

None.

## Funding

Not Applicable.

## Competing interests

None

## Ethics approval

Thesis approval number: 3321.
